# Can innovative ambulance transport avert pregnancy–related deaths? One–year operational assessment in Ethiopia

**DOI:** 10.7189/jogh.06.010410

**Published:** 2016-06

**Authors:** Hagos Godefay, John Kinsman, Kesetebirhan Admasu, Peter Byass

**Affiliations:** 1 Tigray Regional Health Bureau, Mekele, Ethiopia; 2 Umeå Centre for Global Health Research, Epidemiology and Global Health, Dept of Public Health and Clinical Medicine, Umeå University, 90187 Umeå, Sweden; 3 Federal Ministry of Health, Addis Ababa, Ethiopia; 4 Medical Research Council/Wits University Rural Public Health and Health Transitions Research Unit (Agincourt), School of Public Health, Faculty of Health Sciences, University of the Witwatersrand, Johannesburg, South Africa

## Abstract

**Background:**

To maximise the potential benefits of maternity care services, pregnant women need to be able to physically get to health facilities in a timely manner. In most of sub–Saharan Africa, transport represents a major practical barrier. Here we evaluate the extent to which an innovative national ambulance service in Ethiopia, together with mobile phones, may have been successful in averting pregnancy–related deaths.

**Methods:**

An operational assessment of pregnancy–related deaths in relation to utilisation of the new national ambulance service was undertaken in six randomly selected Districts in northern Ethiopia. All 183 286 households in the six randomly selected Districts were visited to identify live–births and deaths among women of reproductive age that occurred over a one–year period. The uptake of the new ambulance transport service for women’s deliveries in the same six randomly selected Districts over the same period was determined retrospectively from ambulance log books. Pregnancy–related deaths as determined by the World Health Organization (WHO 2012) verbal autopsy tool [13] and the InterVA–4 model [14] were analysed against ambulance utilisation by District, month, local area, distance from health facility and mobile network coverage.

**Findings:**

A total of 51 pregnancy–related deaths and 19 179 live–births were documented. Pregnancy–related mortality for Districts with above average ambulance utilisation was 149 per 100 000 live–births (95% confidence interval CI 77–260), compared with 350 per 100 000 (95% CI 249–479) for below average utilisation (*P* = 0.01). Distance to a health facility, mobile network availability and ambulance utilisation were all significantly associated with pregnancy–related mortality on a bivariable basis. On a multivariable basis, ambulance non–utilisation uniquely persisted as a significant determinant of mortality (mortality rate ratio 1.97, 95% CI 1.05–3.69; *P* = 0.03).

**Conclusions:**

The uptake of freely available transport in connection with women’s obstetric needs correlated with substantially reduced pregnancy–related mortality in this operational assessment, though the design did not allow cause and effect to be attributed. However, the halving of pregnancy–related mortality associated with ambulance uptake in the sampled Districts suggests that the provision of transport to delivery facilities in Africa may be a key innovation for delivering maternal health care, which requires wider consideration.

Despite major international concerns around maternal health and institutional delivery rates [1], little innovative thought has been given to the logistic issues of getting African women in to appropriate institutions in a timely fashion. Expecting rural women in labour to walk several kilometres to a facility, possibly at night and in bad weather, is unrealistic. Thirty years ago a startling but small–scale finding from The Gambia found that there had been no maternal deaths for eight years in a group of small villages where resident midwifery services and immediate access to referral transport had been made freely available, when otherwise 16 maternal deaths might have been expected [2]. Though that innovation was widely considered unscalable and unsustainable, millions of pregnant African women have died in the intervening decades, partly from not being able to reach maternity services [3,4].

The principle that effective transport for obstetric health emergencies is essential is not a matter for debate in most settings. However, that thinking has not translated widely into sub–Saharan Africa, where access to obstetric care remains a major barrier, with a lack of transportation and other infrastructure. Only a few sub–Saharan countries have considered and evaluated the provision of ambulances to facilitate access to obstetric care even in emergencies, for example in Burundi [5], Uganda [6,7], and South Africa [8].

In Ethiopia, the 2011 DHS report found 9.9% of births nationally during the previous five years were delivered at a health facility (10.6% in Tigray Region) and 71.1% of women mentioned lack of transport to a facility as a major barrier (52.4% in Tigray Region) [9]. Just 0.1% of rural households owned any kind of motorised transport [9]. The challenges of increasing institutional delivery rates and access to emergency obstetric care have now been recognised. The Ethiopian government health service is now unique in sub–Saharan Africa in providing four–wheel drive ambulances in every rural District (areas each covering around 150 000 people), and in making the ambulances available on a 24–hour, 7–day basis to transfer any woman in labour or experiencing other obstetric difficulties to appropriate health facilities. Parallel innovation in mobile telephony, which has brought widespread network coverage to rural Ethiopia, completes the picture by providing a means to call ambulances when needed [10]. This highly innovative approach to improving maternity care has been rolled out nationally in Ethiopia since 2012.

A total of 1250 ambulances have been distributed, with at least one ambulance per District and nearly half of Districts (larger ones) getting two. The total investment to achieve this was about US $50 million. Before ambulances were deployed, the Ethiopian Federal Ministry of Health signed an agreement with governments of the nine regional states and the two autonomous city administrations (Addis Ababa and Dire Dawa) to regulate use. The agreement entailed three important commitments. First the regional governments committed to allocate budgets to cover the running costs for the ambulances; second they committed to replacing the ambulances after five years; and third they agreed to make ambulance services available free of charge. Furthermore, a number of town–hall and community meetings were held to inform communities about these commitments. Once the ambulances were delivered, frequent reminders about the commitments were sent out through local mass media. Laws to enact the ambulance service provision and ensure and safeguard the proper utilisation of ambulances have also been passed by the regional cabinets.

These developments in Ethiopia therefore provided a unique opportunity to contribute to filling the current evidence gap on the provision of non–emergency obstetric transport in Africa. An operational assessment of the effectiveness of the innovative ambulance service for transporting women to facilities and averting pregnancy–related deaths was conducted, based on two interlinked data sources. Tigray Regional Health Bureau, in the north of Ethiopia, had previously undertaken a one–year representative randomised population survey of pregnancy–related mortality [11], which could be linked at the local community (*tabia*) level to data on actual ambulance utilisation for obstetric care, as a means of assessing the extent to which ambulance utilisation may have averted pregnancy–related deaths.

## METHODS

The detailed methodology for the pregnancy–related mortality survey design has been presented elsewhere [12]. Briefly, the 34 rural Districts (*woreda*) in Tigray Region form six geographic Zones, and one District per Zone was randomly selected as a stratified sample, covering a population of 843 115. A two–stage retrospective household mortality survey was carried out in mid–2013 by community health staff in the selected Districts, following up deaths among women of reproductive age (15–49 years) over a one–year period (from the ninth month of Ethiopian year 2004 to the eighth month of Ethiopian year 2005, corresponding to 9 May 2012 to 8 May 2013 in the international calendar) using the WHO 2012 verbal autopsy tool [13], and deriving cause of death using the corresponding InterVA–4 model [14]. The date, place and personal details were recorded for all deaths. The same survey captured the corresponding number of live–births. Maternal mortality ratio (MMR) is defined here as pregnancy–related deaths per 100 000 live–births, as adopted by the Demographic and Health Survey programme [9].

As a separate exercise, the vehicle log books for the ambulances in the six randomly selected districts underwent retrospective data capture for the same one–year period. The data for each trip included whether it was connected with a delivery; from which community it originated; patient’s name; distance travelled; destination and date. The completeness of the vehicle logs was verified using the odometer readings for the start and end of every journey in each vehicle, before the journeys specifically relating to women delivering were extracted.

Descriptive statistics were used to characterise the patterns of ambulance transportation in relation to pregnancy–related deaths and determinants including District, month, local area, distance from health facility and mobile telephone coverage. Multivariable Poisson regression modelling was used to assess competing contributions of distance to facility, mobile network availability and ambulance utilisation on pregnancy–related mortality at local area level (typically neighbourhoods of around 5000 people).

## RESULTS

**Figure 1:**
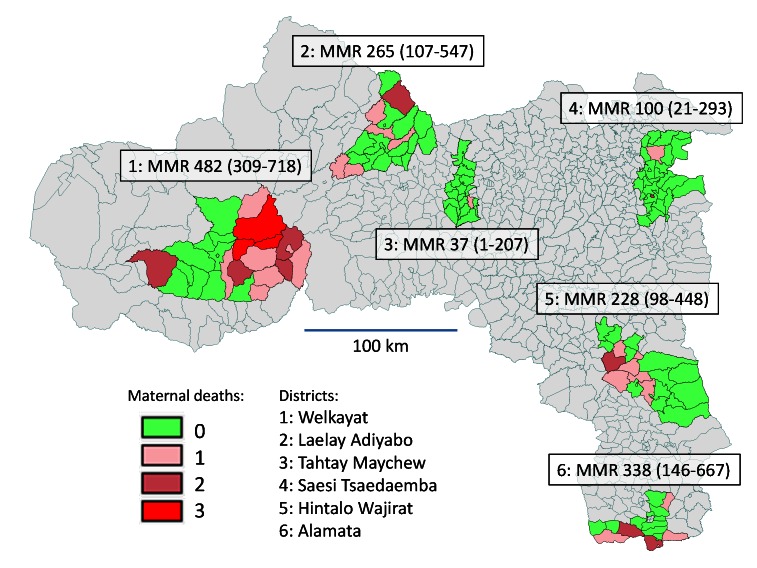
Map of Tigray Region,Ethiopia, showing the six study Districts and maternal mortality ratios (MMR) with 95% confidence intervals for each District. Shading indicates the numbers of maternal deaths in each local area (*tabia*) within each District.

During the one–year period, 51 pregnancy–related deaths and 19 179 live–births were identified in the community survey of 183 286 households in the six study Districts, as previously described [11]. Corresponding data from ambulance log books detailed 4779 trips related to deliveries, covering 178 736 km. **Figure 1** shows MMR in six study Districts within Tigray Region, also showing numbers of pregnancy–related deaths that occurred in each local area during the study period.

**Table 1** summarises births, deaths and MMR across the six study Districts. In two Districts, Alamata and Hintalo Wajirat, ambulances were not yet available at the start of the study, so the parameters for those Districts are shown both for the whole year and, in italics, for only the period during which ambulances were available. The overall percentage of deliveries using ambulances was 24.9%, ranging across the Districts from 6% to 53%. The mean distance per delivery where ambulances were used was 37.4 km, ranging from 23 to 45 km across the Districts. The proportions of ambulance trips going to a hospital rather than a health centre were higher in Alamata District, which contains a hospital, and in Tahtay Maychew District, which is close to Axum hospital.

**Table 1:** Ambulance utilisation in relation to delivery care across six rural Districts in Tigray Region, Ethiopia, over a one–year period

Utilisation	Alamata*	Hintalo Wajirat*	Laelay Adiyabo	Saesi Tsaedaemba	Tahtay Maychew	Welkayat
Live–births	2364 (2021)	3516 (934)	2637	2990	2697	4975
Pregnancy–related deaths	8 (5)	8 (1)	7	3	1	24
MMR per 100 000 live–births	338 (247)	228 (107)	265	100	37	482
Ambulance trips for deliveries	1205	268	496	1590	914	306
% deliveries using ambulances	51.0 (59.6)	7.6 (28.7)	18.8	53.2	33.9	6.2
Ambulance km for deliveries	43 633	12 024	20 574	58 562	36 977	6966
Ambulance mean km per delivery	36.2	44.9	41.5	36.8	40.5	22.8
Ambulance mean km per live–birth	18.5 (21.6)	3.4 (12.9)	7.8	19.6	13.7	1.4
% ambulance trips to hospitals	27.8	1.1	no data	6.7	47.0	4.6

*Figures in brackets for Alamata and Hintalo Wajirat Districts reflect only the part of the year during which ambulances were available.

**Figure 2:**
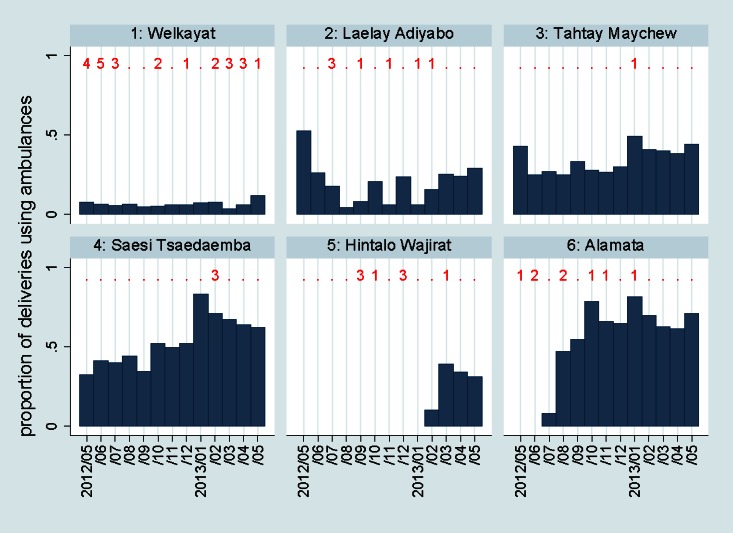
Proportions of deliveries using ambulance transport and numbers of pregnancy–related deaths (figures in red), by month and District.

**Figure 3:**
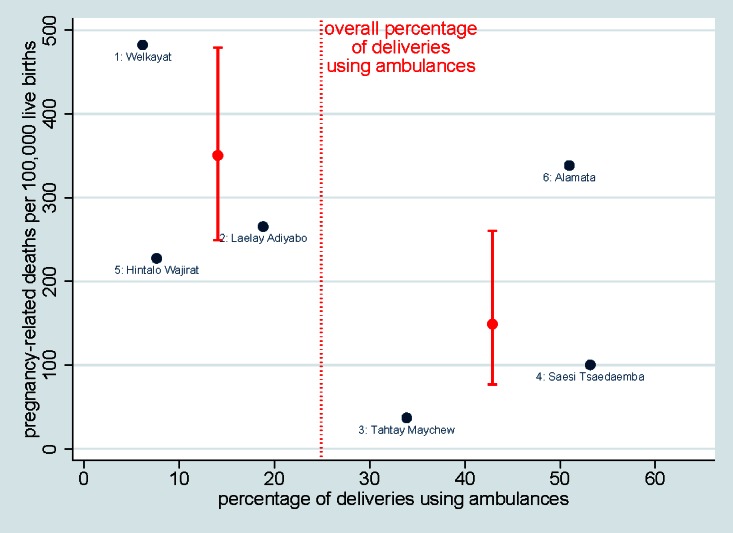
Maternal mortality ratios (MMR) and proportions of deliveries using ambulances for each District. Red bars indicate aggregated MMRs and 95% confidence intervals for Districts achieving above and below the overall percentage (24.9%) of deliveries using ambulances.

The proportion of deliveries using an ambulance was calculated for each month and District as a means of tracking patterns in ambulance utilisation over the one–year period. This is shown in **Figure 2** for each District, together with the numbers of pregnancy–related deaths that occurred in each month. **Figure 3** shows a comparison between Districts above and below the overall 24.9% level of ambulance utilisation. Aggregated MMRs and 95% CIs for the two groups of Districts are shown by the red bars, 350 per 100 000 (95% CI 249–479) and 149 per 100 000 (95% CI 77–260) respectively; *P* = 0.01.

**Figure 4:**
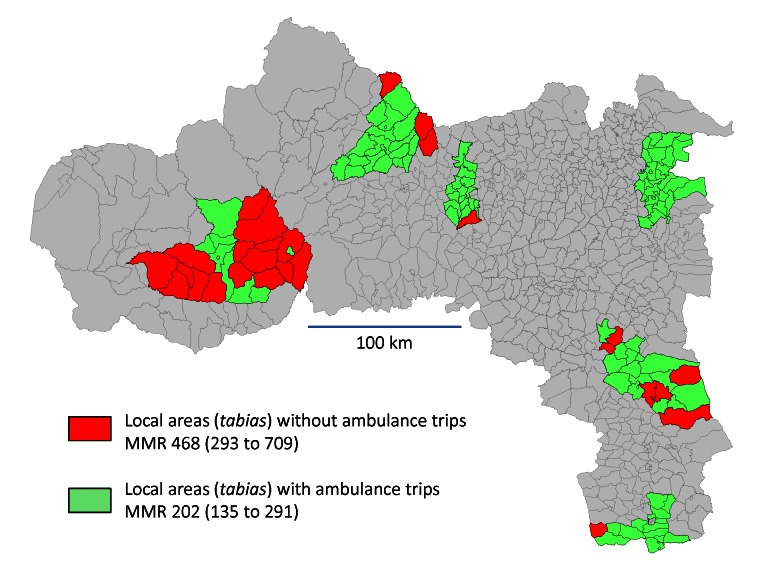
Ambulance utilisation within the six study Districts in Tigray Region, Ethiopia, by local area (*tabia*) and associated maternal mortality ratios (MMR) with 95% confidence intervals.

**Figure 5:**
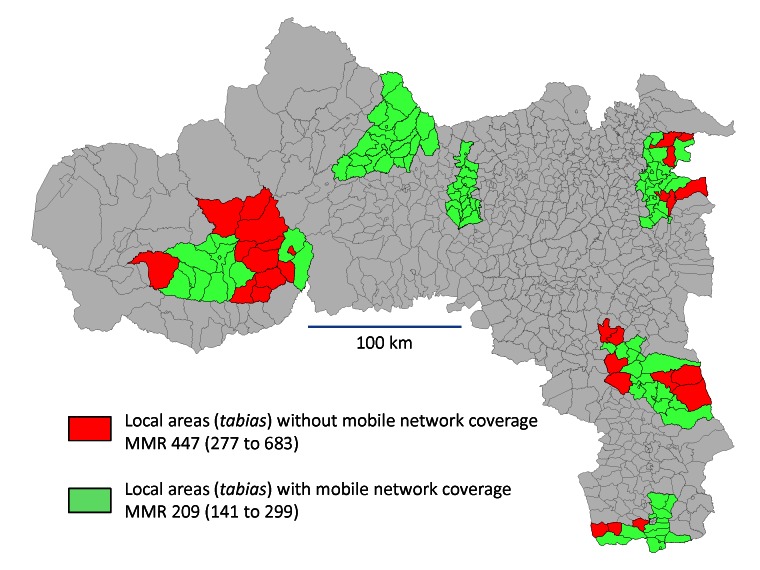
Mobile telephone network coverage within the six study Districts in Tigray Region, Ethiopia, by local area (*tabia*) and associated maternal mortality ratios (MMR) with 95% confidence intervals.

**Figure 4** shows the104/131 local areas utilising ambulance trips in connection with deliveries during the year, together with MMR by utilisation. **Figure 5** shows the 103/131 local areas with mobile telephone network coverage. Pregnancy–related mortality was significantly lower both in the local areas using ambulances (MMR 202, 95%CI 135–291 vs 468, 95%CI 293–709; *P* = 0.006) and in the local areas covered by mobile telephone networks (MMR 209, 95%CI 141 to 299 vs 447, 95%CI 277–683; *P* = 0.014).

A Poisson regression model was constructed including all 131 local areas in the survey, with the number of pregnancy–related deaths as the dependent variable and the number of live–births as the exposure term for each area. Independent variables were distance from the District Health Centre, availability of the mobile telephone network, and ambulance utilisation. **Table 2** shows bivariable and multivariable results from this modelling. In the bivariable model, all the independent variables were significantly associated with pregnancy–related deaths. In the multivariable model, ambulance non–utilisation emerged as the overall significant factor associated with pregnancy–related deaths (mortality rate ratio 1.97, 95% CI 1.05–3.69; *P* = 0.03), while distance to the District Health Centre lost most of its effect.

**Table 2:** Associations between maternal deaths and distance to District Health Centre, availability of mobile telephone network and utilisation of ambulance transport for deliveries in 131 local areas (*tabia*) in Tigray Region, Ethiopia, using a Poisson regression model.

Factor	Level	Number of local areas	Number of pregnancy–related deaths	Bivariable maternal death rate ratio (95% CI)	Multivariable maternal death rate ratio (95% CI)
Distance to District Health Centre	<15 km	32	8	Ref	Ref
	15–30 km	69	23	1.45 (0.65–3.24)	1.23 (0.54–2.79)
	>30 km	30	20	2.33 (1.03–5.28)*	1.15 (0.43–3.11)
Mobile telephone network	Available	103	30	Ref	Ref
	Not available	28	21	2.14 (1.22–3.73)*	1.78 (0.91–3.41)
Ambulance transport for deliveries	Utilised	104	29	Ref	Ref
	Not utilised	27	22	2.31 (1.33–4.03)*	1.97 (1.05–3.69)*

CI – confidence interval

*95% confidence interval excludes unity, *P* < 0.05.

By considering **Figure 3**, it is possible to extrapolate to putative changes in ambulance utilisation in below–average Districts to bring them to above–average levels. The lower group had 1070 utilisations out of 11 128 deliveries, compared with 3709/8051 in the higher group. Thus an additional 3211 deliveries in the lower group would be needed to achieve the same rate of utilisation. Hypothetically assuming that this would have the same effect on pregnancy–related mortality as the observed difference between the lower and higher utilisation groups, a reduction from an MMR of 350 to 149 in the group observed to have lower utilisation would avert 39 × (149/350) = 17 deaths. If each additional ambulance trip involved the mean observed distance of 37.4 km, usage per death averted would be approximately 7000 km. Conversely, therefore, the 178 736 ambulance kilometres that were actually deployed during the study might have averted around 26 pregnancy–related deaths. If that were the case, then the internally adjusted overall MMR for Tigray Region in the absence of the new ambulance service would have been 401 per 100 000 live–births, rather than the 266 per 100 000 observed [11].

## DISCUSSION

These results clearly show substantially lower pregnancy–related mortality in places and periods where free ambulance transport was used by women in connection with their deliveries. We entirely accept that an operational assessment of this kind cannot demonstrate statistically that ambulance utilisation caused reductions in pregnancy–related mortality. Nevertheless, observed variations in pregnancy–related mortality were very substantial, and highly correlated with ambulance utilisation. Since, in most of the world, women’s means of transport to health care facilities for delivery are taken for granted as an essential component of health systems, it is reasonable to suppose that the availability of transport might be just as essential in sub–Saharan Africa.

The physical obstacles to reaching health facilities, and the lack of available transport options, are probably most extreme in Africa. The adjusted MMR estimate of 401 per 100 000 for Tigray in the absence of the ambulance service was consistent with international estimates of MMR for Ethiopia before ambulances were deployed [3], and also similar to MMR survey results from the Southern Nations Nationalities and Peoples’ Regional State in the pre–ambulance period [15]. The lower MMR observed in Tigray when ambulances were available probably accrued from a combination of substantially increasing the proportion of institutional deliveries by transporting women and diminishing transport delays for a smaller proportion of women in acute difficulties. The remote and difficult terrain in Tigray Region also makes it very difficult to undergo transfer to a health facility once complications become evident, and so the provision of a universal service before complications arise may avoid potentially life–threatening transport delays at a later stage. The detailed individual data that would be needed to tease out these different factors were not available.

Ideally this study would have been designed as an intervention with randomised allocation to ambulance transport. However, there would be very serious ethical difficulties in designing such an evaluation, given that the world in general implicitly assumes that availability of effective transportation to access obstetric services is a basic requirement, which may even be considered as a human rights issue [16]. Given the impossibility of mounting a controlled trial, the only available option was to make a transparent operational assessment in the context of the introduction of the new Ethiopian national policy, designed as a retrospective observational study [17]. That said, serendipitous operational delays in ambulance deployment in two of the six Districts, as well as varying effectiveness and coverage by the ambulance service between the randomly selected Districts, enhanced opportunities for comparison.

This operational assessment was only carried out in one Region, where survey data on maternal deaths were already available [11]. Nevertheless it is important to note that Tigray covers some of the most mountainous and hard–to–reach areas of Ethiopia. The ambulance programme in Tigray, by taking a quarter of all delivering women to facilities, made a major impact on previously low institutional delivery rates [9]. Districts included in this operational assessment had been randomly selected for the previous mortality survey [12], and results showed that there were substantial variations between Districts in many parameters. Even though 95% confidence intervals around MMRs are fairly wide in some instances, reflecting the relative rarity of maternal deaths, sufficient numbers were included in the survey to detect important differences. The previous mortality survey and the ambulance log book data capture were undertaken as two completely independent exercises, with neither making use of routine data reporting systems, to ensure integrity and independence. Although retrospective mortality surveys always carry some risk of under–reporting, there is no reason to suppose that any such bias would be correlated with ambulance utilisation. No direct national assessment of pregnancy–related mortality has been made since the ambulance programme was introduced, but our estimates after adjusting for ambulance utilisation reflect pre–ambulance levels as both estimated nationally and as surveyed in another Region [3,15].

Previous studies of obstetric emergency transport in sub–Saharan Africa have been very limited, and none have involved non–emergency provision. A small–scale study in Burundi estimated that a large proportion of cases with complications were transferred by ambulance, but was not able to measure the effect on maternal mortality [5]. Similarly in Ruhira, Uganda, a small study of the use of a single ambulance for emergencies only, with two–thirds of call–outs being for complicated obstetric cases, concluded that it could be cost–effective to provide such a service, but was not able to evaluate changes in maternal mortality [6]. A larger pre– and post– intervention study in Oyam, Uganda, looked at the effect of introducing a single ambulance, and concluded that there was an increased rate of Caesarean sections after the ambulance became available, but also did not evaluate changes in maternal mortality [7]. In Free State, South Africa, the effect of introducing emergency obstetric transport across the Province was evaluated in terms of institutional maternal mortality, with MMR falling from 279 per 100 000 to 152, a risk ratio of 0.54 (95% CI 0.40–0.74) [8], a similar reduction as observed here.

Two factors which might reasonably be supposed to influence pregnancy–related mortality in the absence of a free transport service were distance to District health facilities and the availability of mobile telephone networks. In Tanzania, maternal mortality and distance to hospital were strongly related [18]. In the United States, where availability of transport is presumably not a major issue, distance from hospital has still been shown to be highly associated with maternal deaths [19]. In Oxfordshire, United Kingdom, the use of mobile phones in emergency situations was shown to reduce the risk of death at the scene, though not specifically for pregnancy–related deaths [20]. In the current study, both of these factors could be evaluated on a local area basis, and both individually were highly associated with pregnancy–related deaths, as shown in **Table 2**. However, introducing ambulance use into a multivariable model with these two factors resulted in transport service utilisation emerging as the strongest, and only significant, factor associated with reductions in pregnancy–related deaths. Mobile phone availability retained some of its effect, not surprisingly given that mobile phones are an essential component of the overall ambulance transport policy, providing the only opportunity to call ambulances to communities with no other means of communication. Mobile telephony is a very significant development which has spread across rural Africa relatively recently, and which must be regarded as a huge public health gain. In Ethiopia, EthioTelecom is the sole network provider, with widespread coverage other than in particularly hard–to–reach areas. It may be appropriate for Ministries of Health to put pressure on mobile communications operators to extend coverage into all inhabited areas.

While extrapolating from these results might be speculative, our estimate that approximately 7000 ambulance–kilometres averted one pregnancy–related death suggests that ambulances may be a practical and cost–effective means to substantially reduce the persistently high levels of pregnancy–related mortality across sub–Saharan Africa. Assuming that a free ambulance service such as that now implemented in Ethiopia might cost around US $1 per kilometre to provide (fuel, servicing, depreciation and staff), then an annual budget of US $1 billion for free ambulance transport might avert a substantial proportion of the estimated 180 000 maternal deaths in Africa. Of course this would not simply be a matter of money–resources would have to be translated into a committed and effective transport service, which may not be easy, and local conditions and circumstances would have to be considered. But it seems clear that effective obstetric transport can make a substantial and cost–effective difference to pregnancy–related mortality–possibly achieving as much as two–thirds of the 75% reduction called for by Millennium Development Goal 5 (MDG5) [3].

## CONCLUSIONS

Without doubt the possibility for women to be readily transported to health facilities, both for routine deliveries and in obstetric emergencies, is a critical component of providing effective maternal care. Our results show that transport and communication innovations in Ethiopia correlated with appreciably reduced pregnancy–related mortality. This was achieved through the provision of four–wheel drive ambulances on a 24/7 basis, which could be called via the mobile telephone network. The magnitude of mortality differences correlated with ambulance utilisation amounted to a considerable proportion of the MDG5 target for maternal mortality reduction. Although this assessment only covered one Region in Ethiopia, the magnitude of the observed reduction in pregnancy–related deaths, plus the commonplace notion that women have a right to be able to physically get to maternity services, underlines the urgent necessity of considering the provision of innovative obstetric transport across Africa.
